# Supplementary material for: Node-reconfiguring multilayer networks of human brain function
Source: ArXiv. 2025 Mar 6:arXiv:2410.05972v2. Preprint. [Version 2] (PMC11908370)
Supplement: Supplement 1 [file NIHPP2410.05972v2-supplement-1.pdf]

Supplementary Information for Node-reconfiguring multilayer networks of human brain function

S1 Selection of clustering parameters

Defining optimized ROIs requires setting, depending on the approach, one or two free parameters: the percentage of ROIs used for thresholding and the regularization term in the weighted mean consistency approach, the percentage of ROIs used for thresholding in the minimum correlation approach, and the threshold of the NCUT algorithm. To support the selection of these parameter values, we repeated the clustering with a set of parameter values: ROI percentages 10%, 20%, 30%, 40%, and 50%, regularization parameter values 10, 100, 250, 500, and 1000 and NCUT threshold values 0.1, 0.2, 0.3, 0.4, and 0.5. This yielded 5 different optimized ROI sets per subject and time window for the minimum correlation and NCUT approaches and 25 different ROI sets per subject and time window for the weighted mean consistency approach. We calculated for each ROI set the weighted mean consistency as

$$\phi_{weighted}(I) = \frac{\sum_I^{ROIs} |I|\phi(I)}{\sum_I^{ROIs} |I|} \tag{S1}$$

and the size term

$$S(I) = \frac{\sum_I^{ROIs} |I|^2}{(\sum_I^{ROIs} |I|)^2}, \tag{S2}$$

where the summations go over all ROIs I in the ROI set; these two measures correspond to the two terms of equation 4 of the main text.

Together the weighted mean consistency and size term span a space where the best ROI sets are located close to the top left corner, that is, have large weighted mean consistency and a narrow size distribution indicated by a small size term. To find the parameter combinations that produce such ROI sets, we pooled the weighted mean consistency and size term values of each subject across time windows and constructed the Pareto front, or the set of points where the value of the weighted mean consistency cannot be increased without increasing the size term. Each of the points of the Pareto front corresponds to a selection of parameter values that produces an optimal, albeit different, compromise between maximizing the weighted mean consistency and minimizing the size term. The parameter values selected for the main analysis (ROI percentage 30%, regularization parameter 100, NCUT threshold 0.2) belong to the Pareto front of a vast majority of subjects (Fig. S1; Craddock 24/25, Weighted mean cosnistency 23/25, Min correlation 25/25). Note that other parameter combinations belonging to the Pareto front could have further increased the weighted mean consistency of ROIs. However, as the selected values already yielded higher consistency values than the Brainnetome parcellation, we decided to prioritize narrow size distribution indicated by the low size term.

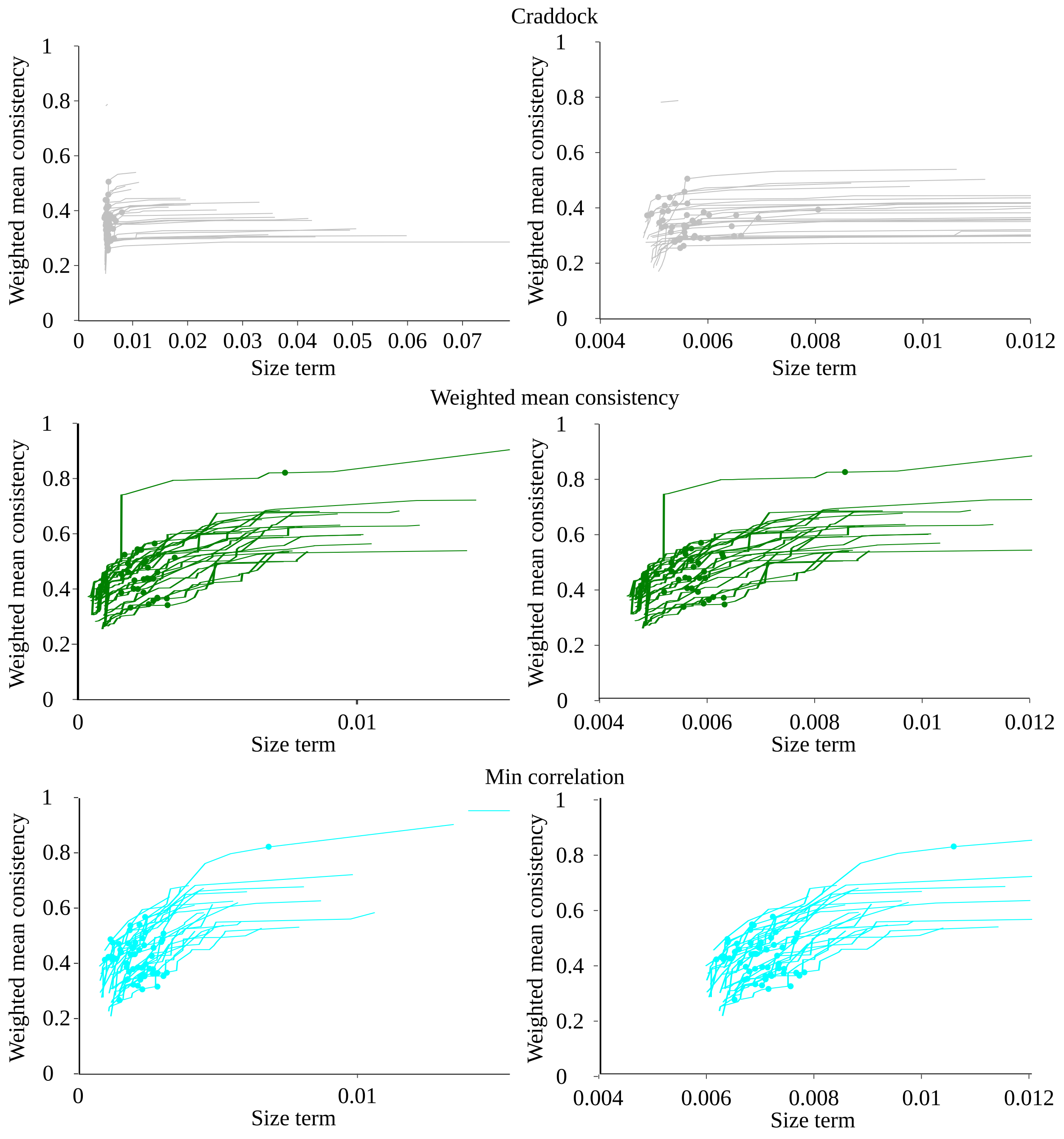

Figure S1: Pareto fronts guide the selection of parameter values. Each line corresponds to the Pareto front calculated for one subject across all time windows for the Craddock (top row), weighted mean consistency (middle row), and minimum correlation (bottom row) parcellation approaches. Markers correspond to parameter values used in the main analysis. Left column shows the full Pareto fronts, while right column shows a zoom-in to the small size term values.

S2 Spatial consistency dynamics

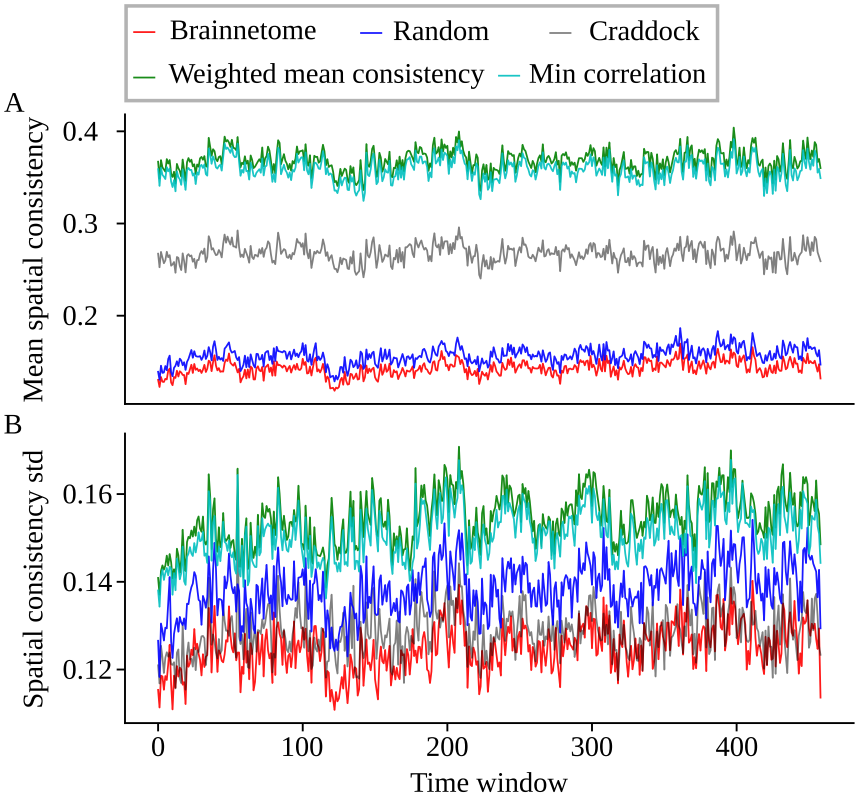

Figure S2: Spatial consistency dynamics. A) Spatial consistency varies in time in all investigated parcellations. However, the changes are relatively small, and no systematic changes or drift are visible in mean spatial consistency calculated across subjects and ROIs. This is not surprising for two reasons. First, systematic, simultaneous changes in multiple subjects are less probable during a naturalistic stimulus then during a stimulus with block design. Second, the optimized parcellation approaches aim for stable, high spatial consistency and let ROI boundaries change to reach this aim. B) The standard deviation of spatial consistency is relatively high in all parcellations, and does not change systematically in time. For visualization purposes, the imaging runs are attached one after another, and time windows are numbered with a continuous index across runs.

S3 Effect of subcortical areas

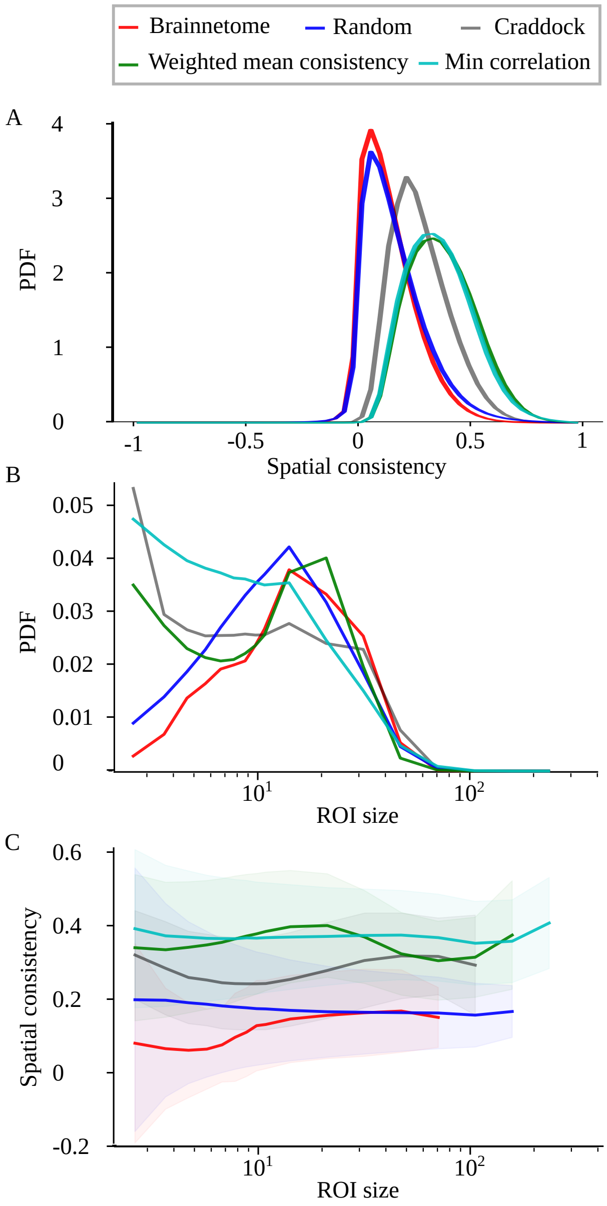

Figure S3: The low spatial consistency of Brainnetome ROIs is not due to subcortical areas. A) Distribution of spatial consistency. B) Distribution of ROI size (defined as the number of voxels in the ROI). C) Average spatial consistency (line) and its standard deviation (shaded area) as a function of ROI size. All distribution has been calculated over all subjects, runs, time windows, and ROIs, excluding ROIs that contain only a single voxel. In C, spatial consistencies have been binned based on ROI size and then bin-averaged.

S4 Spatial consistency with boundaries defined at time t

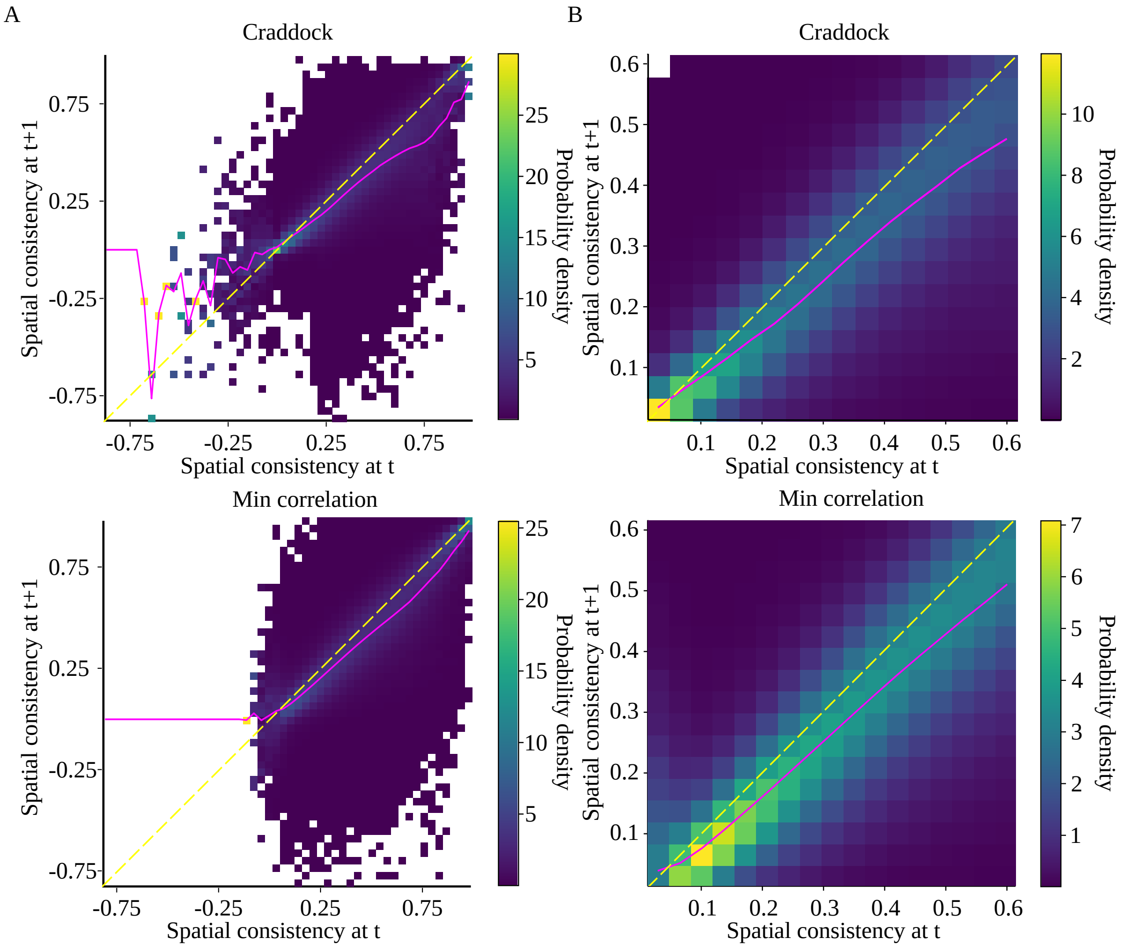

Figure S4: Heatmaps of spatial consistency in time windows t and t + 1 for parcellations produced by the Craddock algorithm and the minimum correlation approach across the whole consistency range (A) and zoomed so that at least 90% of data is included for all parcellations (B). Consistency is calculated with ROI boundaries defined in window t for both windows, and consistency distribution is calculated for each horizontal bin. Magenta line shows the average spatial consistency in each x bin, and yellow dashed line corresponds to $x = y$. White cells contain no data.

S5 Amounts of fully stable ROIs

There are fully stable ROIs in every ROI definition method. The size distributions of these ROIs are presented in Table S1.

	ROI size (voxels)				
Parcellation	1	2	3	4	5+
Brainnetome	2 582	11 691	29 595	51 948	2 671 099
Random	43	35	44	32	82
Craddock	3	6 476	2 182	1 184	3 701
Weighted mean consistency	82 396	15 468	8 100	4 676	9 842
Min correlation	81 070	20 197	11 002	6 066	10 500

Table S1: Number of ROIs with stability score exactly 1 (extreme right of Figure 6B in main text) with respect to ROI size.
